# Phase I of the Detecting and Evaluating Childhood Anxiety and Depression Effectively in Subspecialties (DECADES) Study: Development of an Integrated Mental Health Care Model for Pediatric Gastroenterology

**DOI:** 10.2196/10655

**Published:** 2018-09-10

**Authors:** Stephanie E Hullmann, Stacy A Keller, Dustin O Lynch, Kelli Jenkins, Courtney Moore, Brandon Cockrum, Sarah E Wiehe, Aaron E Carroll, William E Bennett Jr

**Affiliations:** ^1^ Division of Psychology, Department of Psychiatry and Behavioral Sciences Indiana University School of Medicine Indiana University Indianapolis, IN United States; ^2^ Center for Pediatric and Adolescent Comparative Effectiveness Research Department of Pediatrics Indiana University School of Medicine Indianapolis, IN United States; ^3^ Patient Engagement Core Indiana Clinical and Translational Sciences Institute Indiana University Indianapolis, IN United States

**Keywords:** qualitative research, patient-reported outcomes, depression, anxiety

## Abstract

**Background:**

Children with gastrointestinal symptoms have a very high rate of anxiety and depression. Rapid identification of comorbid anxiety and depression is essential for effective treatment of a wide variety of functional gastrointestinal disorders.

**Objective:**

The objective of our study was to determine patient and parent attitudes toward depression, anxiety, and mental health screening during gastroenterology (GI) visits and to determine patient and parent preferences for communication of results and referral to mental health providers after a positive screen.

**Methods:**

We augmented standard qualitative group session methods with patient-centered design methods to assess patient and parent preferences. We used a variety of specific design methods in these sessions, including card sorting, projective methods, experience mapping, and constructive methods.

**Results:**

Overall, 11 families (11 patients and 14 parents) participated in 2 group sessions. Overall, patients and their parents found integrated mental health care to be acceptable in the subspecialty setting. Patients’ primary concerns were for the privacy and confidentiality of their screening results. Patients and their parents emphasized the importance of mental health services not interfering with the GI visit and collaboration between the GI physician, psychologist, and primary care provider.

**Conclusions:**

Patients and their families are open to integrated mental health care in the pediatric subspecialty clinic. The next phase of the DECADES study will translate patient and parent preferences into an integrated mental health care system and test its efficacy in the pediatric GI office.

## Introduction

The Detecting and Evaluating Childhood Anxiety and Depression Effectively in Subspecialties (DECADES) study seeks to develop a model for integrated mental health care that is acceptable to pediatric gastroenterology patients and their families and to compare this model of care with standard care. The first phase of this study sought to develop an integrated mental health care model that is acceptable to both gastroenterology patients and their parents by exploring their attitudes and preferences using qualitative methods augmented by patient-centered design methods.

Depression and anxiety are 2 of the most common disorders occurring during childhood and adolescence [1-3], but they frequently remain unrecognized or untreated [4-6]. Rates of depression and anxiety are significantly higher in children with chronic illnesses [7,8], including gastrointestinal disorders [9-11], than those in the general population. Furthermore, children with depression or anxiety are far more likely to have somatic complaints and greater utilization of subspecialty care, especially in gastroenterology [12-14]. Efforts to recognize and treat mental health problems in children with chronic medical illness, such as gastrointestinal disorders, have been shown to improve adherence to therapy and other clinical outcomes [15,16]. More importantly, improving these mental health concerns may improve the outcomes patients care about the most.

Validated tools exist to screen for anxiety and depression in children, including the Screen for Childhood Anxiety Related Emotional Disorders (SCARED) [17] and the Patient Health Questionnaire (PHQ) [18]. Despite the established importance of depression and anxiety in the gastrointestinal health of children, few data-driven studies exist that describe the identification and management of anxiety and depression by pediatric gastroenterologists and how families or patients view the subspecialty office as the setting to detect or care for mental illness.

Patient engagement is a process by which patients, families, and health professionals work in partnership to improve health care [19], and it is a process for developing patient-centered care. When patients are engaged in the development of new models of care, it improves recruitment and retention to randomized clinical trials, and clinical care is more meaningful to patients and their families [20].

This study describes the development of an integrated mental health care model for pediatric gastroenterology as part of the larger DECADES trial. We sought to develop this model of care using patient-centered design methods to augment qualitative methodology and by directly engaging patients and their parents in the design process. The goals of this study are to determine patient and parent attitudes toward depression, anxiety, and mental health screening during gastroenterology visits and their preferences for communication of results and referral to mental health providers after a positive screen.

## Methods

### Group Sessions

**Figure 1:**
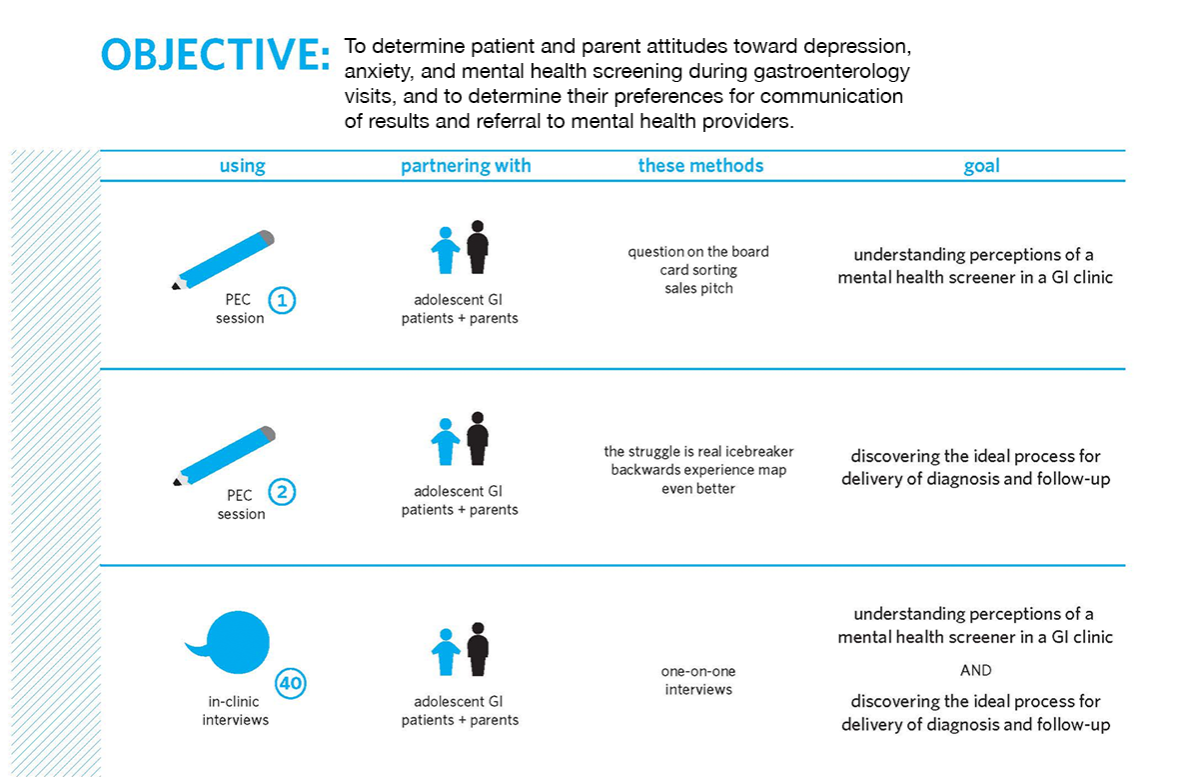
Schematic of the Qualitative Phase of the DECADES Study. GI: gastroenterology; PEC: Patient Engagement Core.

This study involves a series of qualitative group sessions in which standard qualitative focus group methodology has been augmented by patient-centered design methods. At the end of these sessions, we sought to develop a set of specific, actionable recommendations that could then be used to improve patient-centeredness in a subsequent randomized trial. As noted in Figure 1, the overall objective of the qualitative phase of the DECADES study was to develop a greater understanding of patient preferences related to mental health screening in a pediatric subspecialty office. This was accomplished by both individual interviews (the subject of a separate manuscript) and group sessions (the subject of this manuscript).

Patients were seeking care in a pediatric gastroenterology clinic and their parents were approached for enrollment in this qualitative study. Inclusion criteria were as follows: age 5-18 years, a parent or guardian who agreed to participate, and no diagnosed cognitive disabilities. Recruitment was conducted in the pediatric gastroenterology outpatient clinic at Riley Children’s Health, part of Indiana University Health, in Indianapolis, IN. The principal investigator or study coordinator recruited all eligible patients. Permission to approach the patient was obtained from the gastroenterologist of record. Both new and established patients were enrolled.

### Overview of Group Sessions

Two group sessions were conducted with multiple families. Sessions were facilitated by design research specialists using patient-centered design research methods, which are enumerated below, and are based on established methodology in the design literature. Sessions lasted approximately 90 minutes and were audio recorded and transcribed for analysis. All families were compensated for travel to sessions and given a US $30 gift card.

Group sessions used generative design research activities to engage the patients and their parents in codesigning the integrated mental health process [21-23]. Generative design activities tend to be open-ended and allow for a wide range of responses and response types to minimize bias and allow families to be as truthful as possible about their preferences. Sessions began with warm-up activities to encourage participation and collaboration [24]. We then used the following 2 specific types of generative design: projective methods, which are specifically designed to encourage participants to express their thoughts and feelings, and constructive methods to help with concept development [21].

### Specific Patient-Centered Design Activities During Group Sessions

#### Question on the Board

The goal of this activity [24] was to establish participants’ baseline knowledge of anxiety and depression and understand how they express these concepts in their own words, informing how to present a screening tool to patients and their families. Participants were asked to answer the following questions on separate notecards: “What does depression mean to you?” and “What does anxiety mean to you?” Notecards were collected by study staff, and responses were not shared with the group.

#### Card Sorting

The purpose of the card sorting activity [25] was to identify concerns with the screener to help improve how we delivered the screener to patients. For this activity, families were divided into 2 groups (parents and patients) in separate rooms. Both groups were presented with the same stack of cards. Each card listed an item from brief versions of the SCARED and PHQ, the five-item SCARED-5 [17] or the four-item Patient Health Questionnaire [18]. Parents were asked to divide cards into the following 2 piles: “I would be concerned if my child said ‘yes’” and “I would not be concerned if my child said ‘yes.’” Patients were also asked to divide the cards into the following 2 piles: “I would have a hard time answering honestly” and “I would not have a hard time answering honestly.” Next, parents and patients were asked to imagine that they or their child answered all cards in pile #1 affirmatively, and they were asked to write what they would be concerned about happening next and how they would want the results communicated to them. Then, the facilitator encouraged further discussion and elaboration.

#### Sales Pitch

The purpose of the sales pitch was to use projective methods [21] to inform the most acceptable sender, message, and environment for the mental health screener. Both patient and parent participants were asked to convince the person sitting next to them to take the anxiety and depression screeners. Then, they were asked to convince the person to be honest while taking the screener. After completing this exercise, the facilitator encouraged participants to discuss who they would like to explain the screener to them and where they would like to answer the screening questions. They also discussed what steps patients and their parents expected would occur if the patient’s responses yielded a positive screen as well as who would communicate the results of a positive screen.

#### The Struggle is Real

This projective technique was a cartoon completion test [26] used to define what “feeling better” means to patients and their families with regards to anxiety, depression, and gastrointestinal symptoms. Patients and parents sat at separate tables for this activity. Patients were presented with several recognizable memes and were asked to fill in the blanks and react to prompts, such as “My face when...” or “That feeling when...” Parents were presented with 3 different cartoon drawings with blank speech balloons. The first cartoon displayed a frowning child and neutral adult, the second showed a frowning child and happy adult, and the third showed a happy child and a happy adult. Parents were asked to fill in the speech balloons to describe a situation related to having and managing gastrointestinal disorders. After completing the activity, the facilitator encouraged participants to share their responses among the group and facilitated discussion.

#### Backward Experience Map

The backward experience map activity was intended to explore patients’ preferred experience from the time they submit the screening questions to symptom improvement a year later [27]. Patients and parents completed this activity separately. There was a large sheet of paper on the wall with 7 equidistant points connected by an “s”-shaped curve. The beginning point and the last 3 points were subsequently identified (ie, “leaving,” “three months later,” and “one year later”). Participants were asked to identify steps toward getting “better” and fill in the appropriate points on the map. By identifying points that allow participants to get from point A to B, patterns begin to emerge. These patterns begin to uncover themes that establish patient preferences for the treatment experience, patient-centered outcomes, and what “better” means to them.

#### Even Better

This activity used constructive methods [21] to define patient and parent preferences for the best possible sequence of events. Patients and parents completed this activity separately. Expanding upon the results of the backward experience map, participants mapped out their ideal integrated mental health clinic flow process. The facilitator initiated discussion by asking participants to determine what would be “even better” than the ideas that were generated during the backward experience map.

### Analysis

The results of all group session activities were analyzed and coded by the design research specialists who conducted the sessions. They synthesized data from pictures and written documentation (eg, note cards and maps), and they reviewed the audio recordings of the sessions. Data were organized into themes based on Ackoff’s theory [28], which uses a grounded theory approach to distinguish between 3 levels of sense-making (data, information, and knowledge). This study was approved by the Indiana University Institutional Review Board. All patients and family members who participated signed informed consent or assent documents prior to participation.

## Results

### Participants

Overall, 11 families participated in the group sessions, which included 11 patients and 14 parents (Table 1); 5 families participated in the first group session and 6 families participated in the second group session. One family was present for both group sessions.

### Depression and Anxiety

Textbox 1 displays participants’ responses to the question on the board activity. With regard to depression, they described emotional feelings of sadness, negative thoughts (eg, worthlessness), and behaviors consistent with depression (eg, social isolation and withdrawal). Participants described symptoms of various anxiety subtypes (ie, generalized anxiety and social anxiety) as well as physiological symptoms of anxiety (ie, tachycardia, sweating, and nausea).

#### Mental Health Screening and Consultation in the Gastroenterology Clinic

It was important to patients and their parents that patients still receive the gastroenterology (GI) care they intended to receive. Many participants stated that they could be traveling quite a distance for their appointment, and they stressed the importance of keeping their regularly scheduled appointment. One patient explained, "You should still have the GI appointment because that’s what you were scheduled for and you still need that service."

They agreed that if the patient screened positive for depression or anxiety, they would like to discuss it with their doctor and consult with a psychologist, but it was important to them that this discussion did not interfere with their GI appointment.

### Patient Comfort with Mental Health Screening

Patients indicated that their level of comfort with completing mental health screening was related to how the screener was presented. In turn, their comfort would impact how honestly they would answer the questions. If patients felt a sense of control, they would be more likely to respond honestly; for example, patients indicated that if their parents were worried or if the screener was presented unexpectedly with no explanation, they would be more anxious about completing the screener. As a result, patients felt that they may rush through the screening questions or select the most desirable responses. Patients were clear that they would like to be prepared, and they requested to know how many questions are on the screener, how long it would take them to complete, and what would happen after taking the screener.

### Privacy

There was disagreement between parents and patients regarding the privacy of patients’ screening results. When asked about whether parents should receive the results of the screener at the same time as their children, many parents acknowledged that their children would probably want privacy. However, because it is a health issue, parents wanted to be involved and aware of results. Most parents agreed that they had a right to their child’s protected health information; therefore, they screening results should be shared with them. On the other hand, they acknowledged that their children may be less likely to answer questions honestly if they knew their parents would see their results.

Some of the questions might be questionable. They may not want the parent to see. It’s their privacy.

Parent 1

Right, but kids don’t have that yet.

Parent 2

**Table 1:** Group session participant demographic characteristics.

Participants			Session 1	Session 2
**Patient**			**N=5**	**N=6**
	Gender (female), n (%)		4 (80)	5 (83)
	Age (years), mean (SD); range		15.8 (2.7); 11-17	13.8 (3.3); 9-17
	**Race, n (%)**			
		Caucasian	5 (100)	5 (83)
		African American	0 (0)	1 (17)
		Asian	0 (0)	0 (0)
	**Ethnicity, n (%)**			
		Hispanic	0 (0)	0 (0)
		Non-Hispanic	5 (100)	6 (100)
	**Primary gastroenterology complaint, >n (%)**			
		Irritable bowel syndrome	1 (20)	3 (50)
		Abdominal pain	0 (0)	1 (17)
		Vomiting	0 (0)	1 (17)
		Crohn disease	1 (20)	0 (0)
		Eosinophilic esophagitis	1 (20)	1 (17)
		Peptic esophagitis	1 (20)	0 (0)
		Celiac disease	1 (20)	0 (0)
	**Insurance type, n (%)**			
		Commercial	1 (20)	2 (33)
		Public	3 (60)	4 (67)
		Self-pay	1 (20)	0 (0)
**Parent**			**N=6**	**N=8**
	Gender (female), n (%)		5 (83)	7 (88)
	**Race, n (%)**			
		Caucasian	6 (100)	4 (50)
		African American	0 (0)	1 (13)
		Asian	0 (0)	0 (0)
		Unknown	0 (0)	3 (38)
	**Ethnicity, n (%)**			
		Hispanic	0 (0)	0 (0)
		Non-Hispanic	6 (100)	5 (63)
		Unknown	0 (0)	3 (38)

**Participant definitions of depression and anxiety.**

Patient definitions of depression

- “Feeling worthless, being alone.”
- “Living day to day feeling sad and not being able to function in the real world as you would like.”
- “Sadness.”
- “You’re 2 different people, the person on your good day and the person on your bad days.”
- “Being sad.”
- “Feelings of despair. Feeling like nothing goes right.”

Parent definitions of depression

- “An illness of the mind.”
- “Inability to shake ‘the blues,’ feeling there’s something wrong with you that the rest of the world doesn’t ‘get’—that you don’t fit in.”
- “A debilitating mental abnormality as defined by the majority of psychologists & with my experience I would agree.”
- “A feeling of being lonely, wanting to be by yourself, wanting to be left alone”
- “Going to a very dark place in your life. Always feeling sad, not wanting to go anywhere or do anything but sleep all day.”

Patient definitions of anxiety

- “Strong feelings of fear that cause someone to lose normal rational behavior in extreme cases. Nervous or scared.”
- “Shaking”
- “You worry about everything, even the smallest details that don’t matter.”
- “Struggling to go into a large crowd.”
- “Having the feeling in the pit of your stomach that makes you feel nauseous. Fast heartbeat.”
- “Feelings to get through an event where you can’t breathe, have sweats, feelings of being overwhelmed.”

Parent definitions of anxiety

- “Where you don’t want to be in a room with a bunch of people.”
- “Tachycardia.”
- “Also a debilitating mental abnormality, but I think of it more as worrying more often than you need.”
- “Worrying, to the point that the stress caused by worry can sometimes become debilitating. A tight feeling in the pit of your stomach that just won’t go away.”

Most patients did not want their parents to be aware of their screening results unless there was a serious concern. They described being more comfortable discussing these issues with their doctor than with their parents. This was also observed during group sessions. When the parents and patients were together for discussion, patients generally did not express their opinions, but when they were separated from their parents, patients contributed their opinions and actively engaged in discussion. However, there were a few patients who noted that they would feel more comfortable discussing screening results with a parent in the room. One patient explained, "I just feel more comfortable with people I know around."

In both sessions, patients expressed that sharing screening results with their parents may make their parents anxious, and they did not want to worry them. One patient said, "I wouldn’t want her to have to deal with something unless it was like big or something...If I hadn’t told her about that, I wouldn’t necessarily want her to know."

They acknowledged that not being transparent with their parents about screening results may also cause parents to be distressed. Patients agreed that parental worry was a major concern for them. Overall, they expressed that they wanted a choice about whether their parent was in the room while discussing the screening results.

### Communicating Results of Mental Health Screening

Patients agreed that they would not expect their screening results to suggest that they have anxiety or depression and taking a screener might cause some distress for them. Having conversations with their GI doctor and a psychologist would ease their worry about the screener. Most patients wanted their doctor(s) to talk to them without their parents right after taking the screener. All patients stated that they would like some kind of result and plan immediately after taking the screener. Although all patients said they would want some sort of feedback that day, only one said that they would like to get a diagnosis from the screener. Most patients wanted to talk to their GI doctor and a psychologist at the same time right after taking the screener.

### The Best Clinic Experience

When asked what the order of events should be from the moment they completed the screener, patients and parents had little problem creating a process flow that was agreeable to the other participants within their own group but they had difficulty synthesizing a single agreed upon flow. The steps of the desired clinic flow for patients and their parents are included in Table 2 Steps are numbered chronologically and “even better” steps are listed next to the initially desired step.

### Recommendations for the DECADES Study

Based on these results, our design team formulated the following recommendations, all of which have been incorporated in the protocol for the randomized controlled trial portion of the DECADES study.

**Figure 2:**
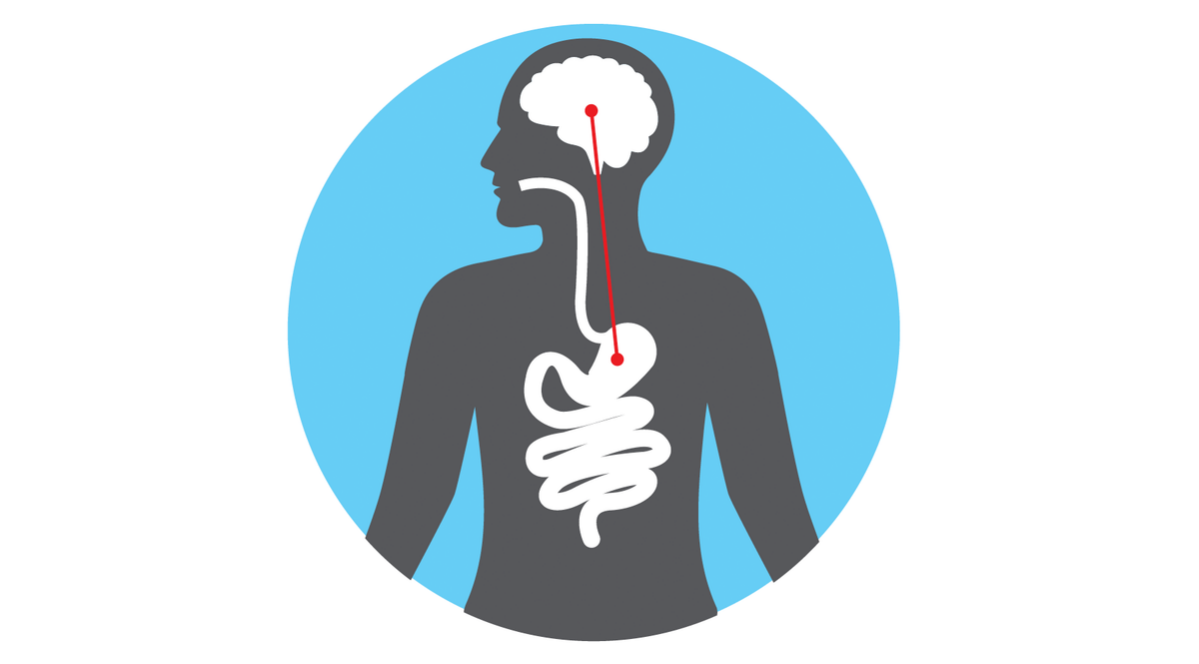
Graphic depicting brain-gut connection.

1. Create a survey or worksheet for the parents to fill out while the patient is taking the screener to both educate and provide a parental distraction
2. Provide a handout that describes depression and anxiety and how it relates to GI symptoms
3. Use informational graphics to educate patients on the relationship between GI symptoms and mental health, as seen in Figure 2
4. Develop an introduction to mental health screening that includes how many questions will be included in the screener, how long it will take, and what will happen after patients complete the screener
5. Use the following language to frame the screener:
  - Regarding the relationship between mental health and physical health: *“FACT: When your GI system is messed up, it can mess with your brain too, causing anxiety or depression. BONUS FACT: When you have anxiety or depression, it can mess with your GI system, causing all kinds of problems.”*
  - Regarding the brain-gut connection: *“Your GI system and your brain—like everything else in your body—are connected. When one is irritated, often so is the other.”*
  - Regarding privacy: *“The answers you give are CONFIDENTIAL. That means they can only be viewed by you and your doctor, unless YOU choose to share it.”*
6. Help patients develop a plan for care that addresses both their mental health concerns and their GI symptoms
7. Talk to the patient separately from the parent and ask patients if they would like their parents to be involved in the discussion
8. Have the patient meet with the GI physician and the psychologist at the same time initially; for example, the physician might say, *“This is (psychologist). She’s going to talk to you about the results of that screener you took. We’ll work together to make a plan for treatment of your depression or anxiety and how they might affect your GI issue.”*

**Table 2:** Steps of desired clinic flow according to patients and parents.

Step	Patient		Parent	
	Desired flow	“Even better” flow	Desired flow	“Even better” flow
1	Immediate feedback from the screener with animation	N/A^a^	Educational information about brain-gut connection, depression, and anxiety available in the waiting area and food provided	N/A
2	Discussion of results with GI^b^ physician	N/A	GI physician gives the results of the screener	Parents and children receive screening results simultaneously; young children may even receive results from their parent
3	GI visit with physician	N/A	Provide additional educational materials	N/A
4	Choose whether or not parents are involved in conversation about screening results	Choose the therapist	Meet with the psychologist and GI physician together	Receive referral to a qualified psychologist close to home. Even better than that would be to be taught coping strategies to use until next appointment.
5	Reassurance and normalization of symptoms	N/A	The family, psychologist, and GI physician agree on a care plan.	Child is involved in care planning.
6	Patients, parents, and care team develop a treatment plan	N/A	Family and care team have a clear understanding of patient’s illness	N/A
7	GI symptom improvement	Being cured of GI symptoms	N/A	N/A
8	Self-management of symptoms with fewer visits to health care providers	N/A	N/A	N/A

^a^N/A: not applicable.

^b^GI: gastroenterology.

## Discussion

We conducted a qualitative study of patient and parent preferences regarding integrated mental health care in the GI office using patient-centered design methods to augment typical group session methodology. Our results suggest that this screening process is highly acceptable to patients and their families with the caveat that confidentiality remains intact, message delivery be customized to the patient or family member, and mental health services do not interfere with their GI visit.

This represents the first attempt, to our knowledge, to develop a set of clear criteria for effective mental health screening in a pediatric subspecialty office. These criteria were developed, not by expert consensus of clinicians as is often the case in similar studies, but by directly engaging with patients and their families who are already visiting this clinic. We believe this will result in a far more effective screening process that is much more acceptable to families and that increases the efficacy of subsequent mental health interventions. We plan to test this in the next, randomized controlled trial phase of the DECADES study.

There are several important limitations to this study. First, owing to the relatively small sample size and few male participants, it is difficult to ascertain broad generalizability of these findings. However, we attempted to recruit patients of various ages, gastrointestinal complaints, and insurance types to increase generalizability to our larger clinic population. Second, the design methods used are novel in health-related research, but they have been well-established in service and product design. Third, results may be limited because adolescents were less likely to contribute to the patient-parent group discussions than their parents. However, patients engaged very well in discussion when they met as a separate adolescent group. Furthermore, the total number of subjects participating in the group sessions was low (11 families comprising 11 children and 14 adults), but these numbers are typical for this type of research, and larger groups tend to be less effective. Most of our subjects were female (both children and parents), which we believe reflects the higher rate of comorbid anxiety and depression in female adolescents as well as greater maternal engagement in child health. Finally, the subjects we recruited were a sample of convenience of nonconsecutive patients seen at our pediatric gastroenterology clinic, who were willing to participate in research, and may not represent a random sample of our patient population.

The next step in the DECADES project is to conduct a randomized comparative effectiveness trial. Patients in the gastroenterology clinic will complete depression and anxiety screening in accordance with the results and recommendations of this first phase of the study. Those who screen positive will be presented with their results and randomized to either standard care or consultation with a pediatric psychologist on the same day as the visit.

## Abbreviations

DECADES: Detecting and Evaluating Childhood Anxiety and Depression Effectively in Subspecialties
GI: gastroenterology
PHQ: Patient Health Questionnaire
SCARED: Screen for Childhood Anxiety Related Emotional Disorders

## References

[1] Costello EJ, Mustillo S, Erkanli A, Keeler G, Angold A (2003). Prevalence and development of psychiatric disorders in childhood and adolescence. Arch Gen Psychiatry.

[2] Kessler RC, Walters EE (1998). Epidemiology of DSM-III-R major depression and minor depression among adolescents and young adults in the National Comorbidity Survey. Depress Anxiety.

[3] Polanczyk G, Salum G, Sugaya L, Caye A, Rohde L (2015). Annual research review: A meta-analysis of the worldwide prevalence of mental disorders in children and adolescents. J Child Psychol Psychiatry.

[4] Ani C, Bazargan M, Hindman D, Bell D, Farooq MA, Akhanjee L, Yemofio F, Baker R, Rodriguez M (2008). Depression symptomatology and diagnosis: discordance between patients and physicians in primary care settings. BMC Fam Pract.

[5] Ferreiro F, Seoane G, Senra C (2012). Gender-related risk and protective factors for depressive symptoms and disordered eating in adolescence: a 4-year longitudinal study. J Youth Adolesc.

[6] S S, Nair Cc, L S R (2015). A Study On Prevalence Of Anxiety Disorders Among Higher Secondary School Students. jemds.

[7] Pinquart M, Shen Y (2011). Depressive symptoms in children and adolescents with chronic physical illness: an updated meta-analysis. J Pediatr Psychol.

[8] Ferro MA, Boyle MH (2015). The impact of chronic physical illness, maternal depressive symptoms, family functioning, and self-esteem on symptoms of anxiety and depression in children. J Abnorm Child Psychol.

[9] Engström I (1992). Mental health and psychological functioning in children and adolescents with inflammatory bowel disease: a comparison with children having other chronic illnesses and with healthy children. J Child Psychol Psychiatry.

[10] Hyams J, Burke G, Davis P, Rzepski B, Andrulonis P (1996). Abdominal pain and irritable bowel syndrome in adolescents: a community-based study. J Pediatr.

[11] Fond G, Loundou A, Hamdani N, Boukouaci W, Dargel A, Oliveira J, Roger M, Tamouza R, Leboyer M, Boyer L (2014). Anxiety and depression comorbidities in irritable bowel syndrome (IBS): a systematic review and meta-analysis. Eur Arch Psychiatry Clin Neurosci.

[12] de BA, Sprangers M, Bartelsman J, de HH (1998). Predictors of health care utilization in patients with inflammatory bowel disease: a longitudinal study. Eur J Gastroenterol Hepatol.

[13] Wong W, Lai K, Lam K, Hui W, Hu W, Lam C, Xia H, Huang J, Chan C, Lam S, Wong B (2003). Prevalence, clinical spectrum and health care utilization of gastro-oesophageal reflux disease in a Chinese population: a population-based study. Aliment Pharmacol Ther.

[14] Cunningham NR, Jagpal A, Peugh J, Farrell MK, Cohen MB, Mezoff AG, Lynch-Jordan A, Kashikar-Zuck S (2017). Risk Categorization Predicts Disability in Pain-associated Functional Gastrointestinal Disorders After 6 Months. J Pediatr Gastroenterol Nutr.

[15] Mackner LM, Crandall WV (2005). Oral medication adherence in pediatric inflammatory bowel disease. Inflamm Bowel Dis.

[16] Keerthy D, Youk A, Srinath AI, Malas N, Bujoreanu S, Bousvaros A, Keljo D, DeMaso DR, Szigethy EM (2016). Effect of Psychotherapy on Health Care Utilization in Children With Inflammatory Bowel Disease and Depression. J Pediatr Gastroenterol Nutr.

[17] Birmaher B, Brent D, Chiappetta L, Bridge J, Monga S, Baugher M (1999). Psychometric properties of the Screen for Child Anxiety Related Emotional Disorders (SCARED): a replication study. J Am Acad Child Adolesc Psychiatry.

[18] Kroenke K, Spitzer RL, Williams JBW, Löwe B (2009). An ultra-brief screening scale for anxiety and depression: the PHQ-4. Psychosomatics.

[19] Carman K, Dardess P, Maurer M, Sofaer S, Adams K, Bechtel C, Sweeney J (2013). Patient and family engagement: a framework for understanding the elements and developing interventions and policies. Health Aff (Millwood).

[20] Domecq J, Prutsky G, Elraiyah T, Wang Z, Nabhan M, Shippee N, Brito J, Boehmer K, Hasan R, Firwana B, Erwin P, Eton D, Sloan J, Montori V, Asi N, Dabrh A, Murad M (2014). Patient engagement in research: a systematic review. BMC Health Serv Res.

[21] Martin B, Hanington B (2012). Universal methods of designways to research complex problems, develop innovative ideas, and design effective solutions. Universal Methods of Design: 100 Ways to Research Complex Problems, Develop Innovative Ideas, and Design Effective Solutions.

[22] Prahalad C, Ramaswamy V (2004). The future of competition: Co-creating unique value with customers.

[23] Sanders E, Stappers P (2012). Convivial toolbox: Generative research for the front end of design.

[24] Michalko M (2010). Thinkertoys: A handbook of creative-thinking techniques.

[25] Cataldo E, Johnson R, Kellstedt L, Milbrath L (1970). Card sorting as a technique for survey interviewing. Public Opinion Quarterly.

[26] Bellenger D, Bernhardt K, Goldstucker J (2011). Qualitative research in marketing.

[27] Adaptive Path (2015). Adaptive Path's Guide to Experience Mapping.

[28] Ackoff R (1989). From data to wisdom. Journal of applied systems analysis.

